# Evidence for a Role of Prolactin in Mediating Effects of Photoperiod during the Dry Period

**DOI:** 10.3390/ani5030385

**Published:** 2015-08-19

**Authors:** Heather M. Crawford, Dawn E. Morin, Emma H. Wall, Thomas B. McFadden, Geoffrey E. Dahl

**Affiliations:** 1 Department of Animal Sciences, University of Illinois, Urbana, IL 61801, USA; E-Mail: heather.crawford@gea.com; 2 College of Veterinary Medicine, University of Illinois, Urbana, IL 61802, USA; E-Mail: d-morin@illinois.edu; 3 Department of Animal Science, University of Vermont, Burlington, VT 05405, USA

**Keywords:** dry period, prolactin, photoperiod

## Abstract

Photoperiod manipulation during the lactation cycle alters milk yield, with long days (LDPP) increasing yield in lactation and short days (SDPP) in the dry period improving subsequent yield. Circulating prolactin (PRL) is directly related to day length, with LDPP increasing and SDPP decreasing PRL, respectively. Two blocks of 24 multiparous Holstein cows were used during two consecutive years to test the hypothesis that the mammary response to SDPP is the result of decreased concentrations of PRL in the circulation relative to LDPP. Cows were randomly assigned to one of three treatment groups during the dry period: SDPP, LDPP, or SDPP+PRL. Cows were returned to ambient photoperiod at calving and milk yield and DMI recorded for 120 d and 42 d, respectively. Mammary biopsies were obtained to determine rates of [^3^H]-thymidine incorporation into DNA *in vitro*. Treatment of SDPP cows with PRL caused a rapid increase in systemic PRL that reached concentrations similar to cows under LDPP. The periparturient PRL surge was similar for LDPP and SDPP+PRL cows, but those groups had greater surge concentrations *versus* SDPP. Cows exposed to SDPP produced more milk than LDPP cows, and there was a trend for SDPP+PRL cows to produce more milk than LDPP cows. Milk production was inversely related to the periparturient PRL surge. There was a trend for a treatment effect on mammary cell proliferation with greater proliferation in mammary tissue of SDPP cows relative to LDPP or SDPP+PRL on day −20 relative to parturition. Replacement of PRL to cows on SDPP when dry resulted in milk yield intermediate to cows on SDPP or LDPP, supporting the concept of a link between dry period PRL and yield.

## 1. Introduction

In dairy cows, the dry period is an essential part of the lactation cycle to maximize yield. During the dry period, the mammary gland undergoes an extensive process of cellular remodeling which is essential for achieving optimal milk production performance in the subsequent lactation [1,2,3]. Because milk production potential is a function of the number of mammary epithelial cells in the gland, as well as the secretory activity of those cells [4,5,6], increased milk yield can be observed under conditions that enhance mammary cell number and (or) mammary cell activity. In addition, any factors involved in the regulation of these processes can directly impact mammary function and milk yield [4]. Therefore, the dry period presents an opportunity to impose management strategies that can influence mammary remodeling and enhance milk yield in the subsequent lactation. One example of such a management tool is manipulation of photoperiod. 

Seasonal effects on mammary development and function have been extensively studied, and it is well established that manipulation of day length influences mammary development and milk production in dairy cattle [7]. Exposure of dairy cows to long day photoperiod (LDPP; 16 h light: 8 h dark) during established lactation (at least 37 days in milk among the studies cited) is associated with increased milk production in comparison to neutral or 12L:12D photoperiod [8,9,10,11]. In contrast, exposure to LDPP during the dry period is associated with a decrease in milk yield in the subsequent lactation relative to short day photoperiod (SDPP, 8 h light: 16 h dark) [12,13]. Although the mechanisms underlying this differential response are unclear, it has been reported that relative to LDPP, prepartum exposure to SDPP is associated with increased mammary cell proliferation and decreased mammary cell death [14], as well as decreased concentrations of prolactin (PRL) in plasma [12,15]. In studies where cows were followed postpartum, a 12L:12D photoperiod was imposed for all cows during lactation, thus PRL concentrations did not differ during lactation [12,14]. In addition, increased serum PRL concentrations during LDPP are associated with a decrease in PRL receptor (PRL-R) expression in mammary tissue of calves [16], cows [17] and rats [18]. 

The importance of PRL in regulating mammary development and function in periparturient ruminants has been clearly established [19,20]. In addition, it has been reported that successful mammary development during pregnancy is dependent on a threshold of PRL-R expression [21]. In the context of photoperiod responses of the non-lactating dairy cow, the increase in serum PRL concentrations and reduced PRL-R expression associated with LDPP might lead to a decrease in PRL signaling and impaired mammary development. Conversely, exposure to SDPP and consequent low PRL concentrations and high PRL-R expression might lead to enhanced PRL signaling during the dry period and increased mammary remodeling. Therefore, researchers have focused on PRL signaling as a potential mediator of the milk yield response [14,17,22]. To date, however, there have been no experiments conducted to clearly distinguish between the effects of altered photoperiod and changes in concentrations of PRL in the circulation of dairy cows. 

We hypothesized that the milk yield response of dairy cows to altered photoperiod during the dry period is due to changes in concentrations of PRL in the circulation, and that treatment of SDPP cows with exogenous PRL would eliminate the milk yield response. To test this hypothesis, we imposed an SDPP treatment combined with a subcutaneous osmotic minipump to restore concentrations of serum PRL to levels similar to cows exposed to LDPP. The main objective was to determine the effect of altered photoperiod combined with exogenous PRL administration on concentrations of serum PRL, mammary cell proliferation, and milk production. Effects on dry matter intake (DMI), bodyweight (BW), milk composition, cow health, and calf birth weight were investigated. 

## 2. Materials and Methods

### 2.1. General 

Two blocks of 24 multiparous Holstein cows from the University of Illinois Dairy herd were enrolled in the study during two consecutive years. Use of animals and all procedures in this study were approved by the University of Illinois Institutional Animal Care and Use Committee. Cows were dried off according to the standard University of Illinois protocol. In year one, cows were dried off between 13 May 2003 and 24 June 2003, and calved between 23 June 2003 and 18 August 2003. In year two, cows were dried off between 31 August 2004 and 28 October 2004, and calved between 29 October 2004 and 23 December 2004. Cows were blocked on the basis of expected calving date and 305-d milk production and randomly assigned to one of three treatment groups on the day of dry-off, which occurred approximately 60 d before calving. All four teats were infused with cephapirin benzathine (Cefi-dri, Fort Dodge Animal Health, Fort Dodge, IA) or penicillin and novobiocin (Albadry, Pfizer Animal Health, Kalamazoo, MI) at dry-off. Treatments were SDPP (8L:16D), LDPP (16L:8D), and SDPP+PRL (SDPP plus exogenous PRL administered by s.c. osmotic minipump). In year one, exogenous PRL was administered at a rate of 12 mg/d for the last 28 d of the dry period (actual infusion length was 25.9 ± 7.8 d). In year two, the rate was changed to 16 mg/d for the last 39 d of the dry period to more closely match the PRL concentrations previously observed under LDPP (actual infusion length was 39.6 ± 3.0 d). Photoperiod was controlled throughout the dry period by housing cows in a mechanically ventilated, controlled light freestall barn. For the SDPP and SDPP+PRL cows lights were on between 0800 and 1600 h daily. For the LDPP group, lights were on between 0800 and 2400 h daily. 

Cows were moved to a tie-stall barn within 24 h after calving and maintained under ambient photoperiod conditions during lactation. In year one, calving occurred between June 23 (sunrise 0525 h, sunset 2026 h; approximately 15L:9D) and August 18 (sunrise 0607 h, sunset 1946 h; approximately 13.5L:10.5D). In year two, calving occurred between October 29 (sunrise 0720 h, sunset 1754 h; approximately 10.5L:13.5D) and December 23 (sunrise 0713 h, sunset 1632 h; approximately 9.5L:14.5D). 

Diets fed to cows during the far-off dry period (d −60 to −22), close-up period (d −21 to d 0), and lactation (d 1 to 120) are presented in Table 1. Diets were formulated to meet NRC requirements for dry and lactating cows. Cows were fed individually during the dry period (using a Calan feeding system) and until 42 d of lactation. Feed intake and refusal were recorded daily and combined to produce weekly averages. The dry matter content of diets was analyzed weekly and used to correct feed intake to calculate DMI. Cows were weighed weekly from dry-off until 6 week of lactation during both years. 

**Table 1:** Diet composition and analysis of total mixed rations (TMR) fed to cows in during the dry period and lactation in both years of the study. Cows were exposed to long day photoperiod (LDPP; n = 12), short day photoperiod (SDPP; n = 12), or SDPP + prolactin (SDPP+PRL; n = 13) during a 60-d dry period, and production variables were assessed during the subsequent lactation.

	Far off Diet d −60 to d −22	Close up Diet d −21 to 0	Lactation Diet d 1 to 120
Ingredients % of DM			
Corn Silage	21.1	38.2	24.7
Alfalfa Silage	41.7	6.6	15.6
Alfalfa Hay	--	10.1	7.8
Protein Blend	11.1	45.2	7.1
Wheat Straw	26.2	--	--
Soy Hulls	--	--	4.0
Wet Brewers Grains	--	--	12.3
Cotton Seed	--	--	7.6
Ground Corn	--	--	20.8
Chemical Composition			
Dry matter	54.6	56.0	52.2
Crude Protein	11.9	15.2	17.7
Available Protein	10.9	14.3	16.7
ADICP	1.0	0.8	1.0
Adjusted CP	11.9	15.2	17.7
ADF	38.9	28.3	26.5
NDF	57.0	42.9	39.1
TDN	63.8	68.3	68.9
NEG or NEL (Mcal/kg)	0.81	0.96	1.62
Calcium	0.8	0.9	0.8
Phosphorus	0.2	0.3	0.4
Magnesium	0.2	0.3	0.3
Potassium	1.9	1.5	1.4
Sodium	0.1	0.1	0.4
Iron (PPM)	500	418	279
Zinc (PPM)	83	129	100
Copper (PPM)	15.5	18.5	17.5
Manganese (PPM)	115.5	94.5	83.5
Molybdenum (PPM)	0.6	0.7	0.9

### 2.2. Blood Collection and Assays

Blood samples (10 mL) were collected from each cow between 0830 and 1030 h by jugular venipuncture weekly starting at dry off and continuing until calving. Blood sampling frequency increased to twice daily (0830 to 1030 and 1630 to 1830) as calving neared to more fully characterize the periparturient PRL surge. Blood samples were collected into a sterile evacuated tube containing sodium heparin (Vacutainer^®^, Becton Dickinson and Co., Franklin Lakes, NJ, USA) and placed on ice immediately. Plasma was harvested by centrifugation at (1850× g, 20 min, 4 °C) within 2 h of blood collection. Plasma was stored at −20 °C until PRL radioimmunoassay as described by Miller *et al*. [11]. Mean intra- and interassay coefficients of variation were 3.3% and 11.1%, respectively. Assay sensitivity averaged 0.93 ng/mL.

### 2.3. Non-Esterified Fatty Acids (NEFA) and Beta-Hydroxybutyrate (BHBA) Assays

Blood was collected from the jugular vein for determination of NEFA and BHBA concentrations. The plasma used was prepared in the same method as described in the PRL assay section. Plasma from d −7, 0, and +7 was analyzed for NEFA concentrations with a NEFA C kit (Wako Chemicals, Dallas, TX, USA) with the method adapted by Johnson and Peters [23]. Plasma BHBA concentrations from d −7, 0, and +7 were determined on an autoanalyzer (Hitachi 911, Tokyo, Japan) at the University of Illinois College of Veterinary Medicine diagnostic laboratory [24].

### 2.4. Implants

Prolactin was administered to cows in the SDPP+PRL groups by means of an osmotic minipump (Alzet Osmotic Pumps, Cupertino, CA, USA). Neither positive (SDPP) or negative (LDPP) control cows received minipumps. In year one osmotic minipumps were implanted s.c. 28 d before expected calving date. The skin over the shoulder was clipped and scrubbed and 2% lidocaine was administered s.c. Implants were placed s.c. behind the left shoulder by making an incision with a scalpel blade and using hemostats to create a pocket for the implant. The implant was then placed in the pocket and the incision was closed with staples. Implants were removed after calving using the method described above. Recombinant bovine PRL was kindly provided by Dr. John Byatt (Monsanto Corp., St. Louis, MO, USA). Pumps were calibrated to deliver 12 (year one) or 16 (year two) mg PRL/d using a 0.9% saline vehicle.

Additional blood samples were collected from a subsample of cows to assess circulating PRL following placement of the osmotic minipumps. Samples were collected as described above the day preceding and daily for 7 d following the implant insertion, and again at 14 d after implant to characterize circulating PRL for the expected life of the minipump.

### 2.5. Milk Production and Sampling

Cows were milked twice daily throughout their lactation and milk production was recorded electronically for 120 d postpartum. Daily milk yields were averaged to calculate weekly values. Milk samples were collected weekly for the first 4 week after calving then monthly for the rest of lactation by DHIA. Milk samples were analyzed by Dairy Laboratory Services, Inc., (Dubuque, IA, USA) for compositional analysis (fat, protein, lactose, and SCC). 

### 2.6. Physical Exams

At dry off all cows were given a full physical exam. Weekly abbreviated physical exams were completed until calving. At calving cows were given a full physical exam and calf weights were recorded. After calving daily physical exams were done for 10 d in Year one, and every 2 d in Year two. Subclinical ketosis was defined as measurement with a urine test that was greater than trace (Ketostix; Bayer, Pittsburgh, PA, USA). A retained placenta was diagnosed when the fetal membranes were retained for greater than 24 h after calving. 

### 2.7. Mammary Biopsy

Mammary biopsies were obtained (n = 8 cows/treatment) on d −40, −20, and +7 relative to expected calving (dates relative to actual calving were −43, −21, and +8). Mammary tissue was obtained from the rear quarters of each cow as described by Farr *et al*. [25]. Left rear quarters were biopsied at −40 d, right rear at −20 d, and left rear at +7 d. Briefly, cows were sedated by intravenous injection of xylazine HCl (35 μg/kg of BW, Phoenix Pharmaceuticals, St. Joseph, MO, USA). The region of the udder to be biopsied was clipped and scrubbed three times with an iodine scrub, and then rinsed with 70% ethanol. For local anesthesia, lidocaine HCl (3 mL; 20 mg/kg, Phoenix Pharmaceuticals, St. Joseph, MO, USA) was administered in a line block above the biopsy site. An incision of ~2 cm was made through the skin and gland capsule. A biopsy sample of ~500 mg (~70 × 40 mm in diameter) was obtained, and the wound was closed with 18 mm stainless steel woundclips (Autoclip^®^, Clay Adams, Parsippany, NJ, USA). Biopsy samples were trimmed of extra-parenchymal tissue and the remaining parenchyma was diced into explants that were used to measure incorporation of [^3^H]-thymidine into DNA. 

### 2.8. Mammary Proliferation Assay

Mammary parenchyma was diced into explants and approximately 100 mg was incubated in a shaking water bath for 1 h at 37 °C in 3 mL of medium 199 (Sigma, St. Louis, MO, USA) supplemented with 1 μCi/mL of [^3^H]-thymidine (33 Ci/mmol, ICN, Irvine, CA, USA) to determine incorporation of [^3^H]-thymidine into DNA. After incubation, explants were blotted, weighed, and frozen in liquid nitrogen. Incorporation of [^3^H]-thymidine into DNA was determined using methods described by Wall *et al*. [14]. 

### 2.9. DNA Assay

Total DNA in tissue homogenates was measured as described by Labarca and Paigen [26], but modified for assay in a 96-well plate as described previously [14]. Briefly, duplicate 2 μL aliquots of homogenate were pipetted into wells, and 98 μL of DABS-E (0.5 M dibasic NaPO_4_, 0.5 M monobasic NaPO_4_, 2 M NaCl and 0.1 M EDTA) buffer and 100 μL of 2 μg/mL Hoechst 33528 dye (Sigma, St. Louis, MO, USA) were added. Fluorescence was determined using a fluorimeter spectrophotometer (KC4, Bio-Tek Instruments, Winooski, VT, USA). The DNA concentration of homogenates was determined by comparison to a standard curve made from serial dilutions of calf thymus DNA (Sigma, St. Louis, MO, USA) and was used to calculate the total amount of DNA in the original homogenate. 

### 2.10. Statistical Analysis

Statistical analyses were performed using the SAS System v 9.1 (SAS Institute, Inc., Cary, NC, USA). Birth weight, dry period length, and pregnancy length were analyzed by ANOVA using the GLM procedure of SAS 9.2 (SAS Institute, Cary, NC, USA). A mixed model was used to analyze all repeated measures data; an autoregressive covariance structure was used. Specific comparisons were made for milk production, milk components, DMI, concentrations of PRL, NEFA, and BHBA, BW, and mammary tissue proliferation between treatments across time. A covariate for 305 d ME milk in the previous lactation was included in the model used to analyze milk production. Average daily temperature was used as a covariate in the model for analyzing PRL concentrations. Response patterns across variables were similar in both years, and data were analyzed together with year included in the model. Least square mean and standard error of the mean are reported. GraphPad Prism^®^ was used to determine the correlation (Pearson’s r) between serum PRL at parturition (*i.e.*, surge peak) within a cow and milk production through 120 d of lactation.

## 3. Results

### 3.1. Prolactin 

**Figure 1:**
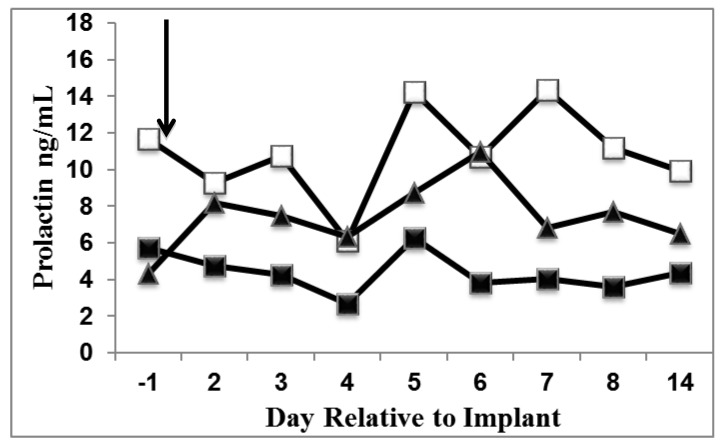
Prolactin (PRL) concentrations near the time of administration of PRL osmotic minipumps to SDPP+PRL (16 mg/d) cows in Year two of the study. Cows were exposed to long day photoperiod (LDPP, n = 8; □), short day PP (SDPP, n = 8; ■), or SDPP+PRL (n = 7; ▲). The black arrow represents when implants were administered to SDPP+PRL cows. From d 2 to 14, LDPP cows had the highest PRL concentrations, followed by SDPP+PRL cows being intermediate, and SDPP cows having the lowest PRL concentrations (*P* ≤ 0.04). SEM ± 1.0 ng/mL.

A sub group of cows (LDPP, n = 8; SDPP, n = 8; SDPP+PRL, n = 7) was used to characterize the effect of photoperiod and PRL replacement on PRL profiles before and after pump insertion and exposure to different lighting schedules during year two. As expected, PRL concentrations did not differ among treatments at dry off (*P* > 0.33). In contrast, after about 14 d exposure to photoperiodic treatment (and 1 d before implant insertion), PRL concentrations of LDPP cows were greater than those of SDPP cows (*P* ≤ 0.03) or SDPP+PRL cows (*P* ≤ 0.01), whereas PRL concentrations of SDPP and SDPP+PRL cows did not differ (Figure 1; *P* > 0.61). After implant placement into SDPP+PRL treated cows, PRL concentrations in LDPP cows were greater than SDPP (*P* ≤ 0.01) and tended to be greater than SDPP+PRL cows (*P* ≤ 0.08), and SDPP+PRL cows PRL concentrations were greater than SDPP cows (*P* < 0.02). Overall during the dry period LDPP cows had higher PRL concentrations than SDPP cows (*P* < 0.01). 

**Figure 2:**
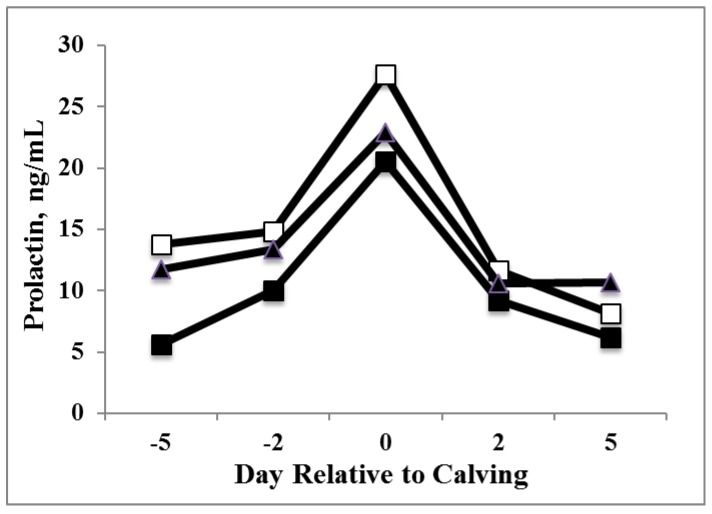
Effects of photoperiod (PP) and prolactin (PRL) treatment on the periparturient PRL surge (Years one and two) in cows exposed to variable PP treatments during the dry period. Treatments included long day PP (LDPP; □), short day PP (SDPP; ■), or SDPP+PRL (▲). Overall LDPP cows had greater PRL concentrations than SDPP cows (*P* = 0.0002), and SDPP+PRL cows had greater PRL concentrations than SDPP cows (*P* = 0.009). SEM ± 0.9 ng/mL.

To examine the effect of photoperiod and PRL replacement on the periparturient PRL surge, concentrations of PRL were combined within groups for samples collected d −5, −2, 0, +2 and +5 d relative to parturition for analysis. Cows treated with LDPP had greater PRL concentrations than SDPP cows (Figure 2; *P* < 0.001); in SDPP cows, PRL concentrations were lower than SDPP+PRL concentrations. Overall there was no difference between PRL concentrations of LDPP and SDPP+PRL cows during the periparturient surge (*P* > 0.15). 

### 3.2. Milk Production and DMI

**Figure 3:**
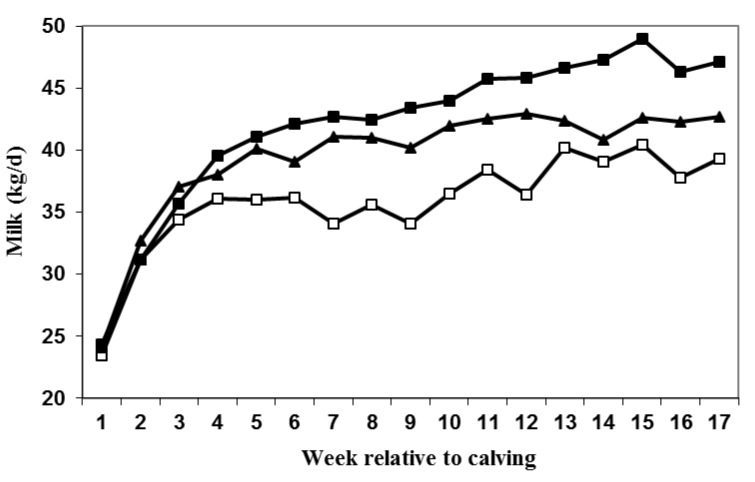
Effect of dry period photoperiod and prolactin (PRL) treatment on subsequent milk production. Cows were exposed to long day photoperiod (LDPP; □), short day photoperiod (SDPP; ■), or SDPP+PRL (▲). Overall SDPP cows had greater milk production through 120 d of lactation than LDPP cows (*P* = 0.06) and SDPP+PRL had intermediate milk production to LDPP and SDPP cows. SEM ± 1.3 kg/d.

**Figure 4:**
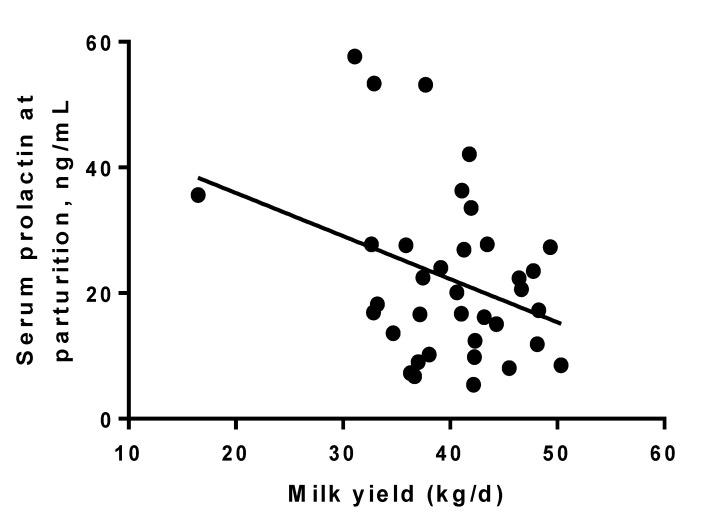
Relationship between periparturient prolactin (PRL) concentrations in the serum and milk production through 120 d of lactation. Differences in PRL were induced by exposure to long day photoperiod (LDPP), short day (SDPP), or SDPP+PRL during the entire dry period. Subsequent milk production was negatively correlated with serum PRL concentrations at the peak of the periparturient PRL surge at parturition (r = −0.33; *P* < 0.05).

Data after calving was lost from 4 cows due to death shortly after calving during Year one, and an additional three cows due to death and three cows due to severe mastitis in the second year of the study. Losses were even among groups with three SDPP, three SDPP+PRL and four LDPP animals being removed from the study. Milk production responses indicated that SDPP (42.6 ± 1.3 kg/d) cows produced more milk than LDPP (38.0 ± 1.3 kg/d) cows whereas yield of SDPP+PRL (40.8 ± 1.3 kg/d) cows tended to be similar to SDPP cows for the first 17 week of lactation when data collection ended (Figure 3). Milk production through 120 d of lactation was negatively correlated with serum PRL concentrations at parturition (r = −0.33; *P* < 0.05; Figure 4). The percentage of milk fat and protein did not differ among treatments (Table 2), but lactose percentage tended to be lower in LDPP cows relative to other treatments. 

A tendency for treatment to alter mammary health, as indicated by somatic cell score (SCS) was observed (Table 2). Overall SCS was lower in SDPP cows compared with LDPP cows (*P* = 0.04), and SDPP+PRL cows tended to have lower SCS than LDPP cows (*P* = 0.08). Overall SCS did not differ between SDPP and SDPP+PRL cows (*P* > 0.73). When somatic cell count (SCC) at dry off and calving were analyzed, dry off SCC were SDPP (509.7), SDPP+PRL (787.4), and LDPP (410.7 SCC/mL × 1000) and did not differ among treatments (*P* > 0.15). At calving LDPP cows had increased SCC (2055.6) compared with their dry off SCC (*P* = 0.003). No other comparisons within treatments were different (*P* > 0.15).

**Table 2:** Milk yield, milk components, somatic cell score, bodyweight, and feed intake for cows exposed to long day photoperiod (LDPP; n = 12), short day photoperiod (SDPP; n = 12), or SDPP+prolactin (SDPP+PRL; n = 13) during a 60-d dry period.

Variable	LDPP	SDPP	SDPP+PRL	SEM	*P*-value
Milk yield, kg/d	38.0	42.6	40.8	1.5	0.09
Fat, %	4.01	3.90	3.88	0.15	0.81
Protein, %	3.13	2.95	3.05	0.1	0.15
Lactose, %	4.63	4.81	4.73	0.08	0.11
SCS	4.33	2.80	3.05	0.5	0.08
BW, kg					
Prepartum	712	717	710	6	0.72
Postpartum	671	669	646	17	0.53
DMI, kg/d					
Prepartum	13.3	14.1	13.1	0.7	0.47
Postpartum	17.5	17.6	16.5	0.7	0.48

There was no effect of treatment on BW before or after calving (Table 2). Likewise, treatment had no impact on DMI prepartum or postpartum (Table 2), although postpartum intake recording was truncated at d 42. 

### 3.3. NEFA and BHBA

In examining NEFA concentrations from d −7 to d +7 relative to calving, concentrations increased in all treatments after parturition (Table 3). There were no differences among treatments at d −7 or 0 (*P* > 0.35; Table 3), yet SDPP cows did have greater NEFA concentrations than LDPP and SDPP+PRL cows at d +7 (*P* = 0.06). There were no differences in BHBA concentrations among treatments either before or after calving (*P* > 0.24).

**Table 3:** Effect of exposure to long day photoperiod (LDPP ^1^), short day photoperiod (SDPP ^2^), or SDPP+prolactin (SDPP+PRL ^3^) during the dry period on circulating concentrations of NEFA and BHBA.

Variable	LDPP ^1^	SDPP ^2^	SDPP +PRL ^3^	SEM	*P*-value
NEFA (UeqL)					
d −7	138	112	105	40	0.36
d 0	413	361	475	40	0.57
d 7	475	673	411	41	0.06
BHBA (mmol/L)					
d −7	0.57	0.66	0.62	0.15	0.24
d 0	0.73	0.74	0.72	0.15	0.24
d 7	0.59	0.75	0.88	0.15	0.36

^1^ NEFA data include LDPP cows from both years (n = 12) whereas BHBA data include only year two (n = 5). ^2^ NEFA data include SDPP cows from both years (n = 12) whereas BHBA data include only year two (n = 6). ^3^ NEFA data include SDPP+PRL cows from both years (n = 13) whereas BHBA data include only year two (n = 6).

### 3.4. Dry Period Length, Calf Weights, and Health

Dry period length was decreased in SDPP+PRL (55 ± 1.8 d) cows when compared to LDPP (60 d; *P* < 0.01) and SDPP (58 d; *P* < 0.07) cows, and gestation length followed a similar pattern. However, treatment did not affect calf birth weight (LDPP = 46.4 ± 2.4 kg; SDPP = 45.5 ± 2.5 kg; and SDPP+PRL = 49.4 ± 2.5 kg; *P* > 0.15). No differences among treatments were observed in the incidence of retained placenta or displaced abomasum. For both years, the incidence of sub-clinical ketosis was 8 of 16 in LDPP cows, 6 of 16 in SDPP+PRL cows, and 10 of 16 in SDPP cows.

### 3.5. Mammary Tissue Proliferation

**Figure 5:**
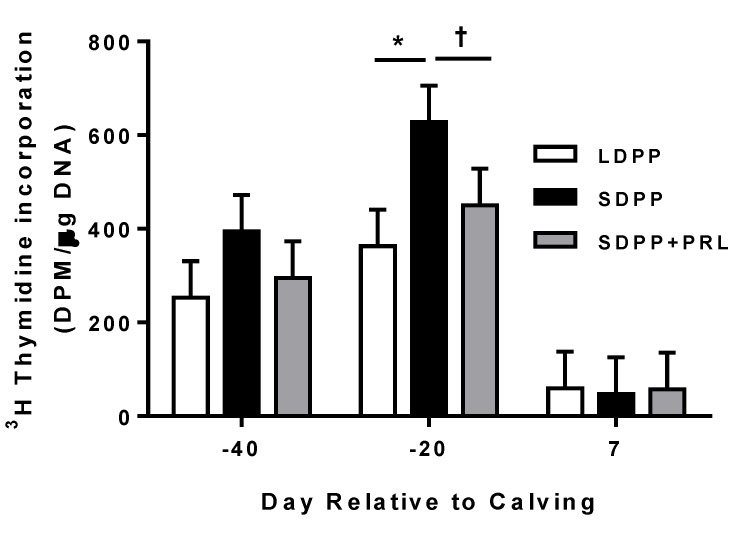
Effect of days relative to calving and photoperiod on incorporation of [^3^H]-thymidine into DNA *in vitro* by mammary tissue of cows exposed to long day photoperiod (LDPP; □), short day PP (SDPP; ■), or SDPP + prolactin (SDPP+PRL; ■) during the dry period. Mammary biopsies were taken on −40, −20, and +7 d relative to parturition. Each bar represents mean ± SEM incorporation (dpm/ug DNA) for cows on each photoperiod at each biopsy (8 cows/treatment). Incorporation of [^3^H]-thymidine into DNA was affected by time relative to calving (*P* < 0.001) and there was a trend for a treatment effect (*P* = 0.12) such that at −20 d relative to parturition, mammary tissue proliferation was increased in SDPP cows relative to LDPP (* *P* < 0.05) or SDPP+PRL (^†^ *P* = 0.15) cows.

Mammary proliferation, measured by incorporation of [^3^H]-thymidine into DNA, increased between −40 d and −20 d, followed by a marked decrease between −20 d and +7 d relative to parturition (overall time effect *P* < 0.001; Figure 5). In addition, we observed a trend for a treatment effect, such that mammary tissue proliferation on day −20 relative to calving was increased in cows exposed to SDPP compared to LDPP or SDPP+PRL cows (Figure 5). At −40 d relative to parturition, incorporation of [^3^H]-thymidine was similar across treatment groups (*P* > 0.20; Figure 5). However at −20 d relative to parturition, incorporation of [^3^H]-thymidine into DNA of cows exposed to SDPP was nearly two-fold greater than those exposed to LDPP (*P* < 0.05; Figure 5) and was nearly 1.5-fold greater than those exposed to SDPP+PRL (a tendency at *P* = 0.15; Figure 5). On day +7 relative to parturition, proliferation of mammary tissue was similar across treatment groups (*P* = 0.80). 

## 4. Discussion

The findings of this study are consistent with previous observations that PRL concentrations are increased in dry cows exposed to LDPP compared with SDPP cows [12,17,27]. In previous experiments, osmotic minipumps were used in steer calves to increase circulating PRL in SDPP treated animals to concentrations similar to LDPP animals, and a concomitant shift in immune function similar to calves on LDPP was observed in SDPP+PRL calves [28]. In part because the supply of recombinant bovine PRL was limited in the present study, PRL concentrations were increased in SDPP+PRL relative to SDPP cows, but lower than LDPP cows. This finite quantity of rbPRL available also limited the number of animals that could be infused, and loss of animals may have limited the power to detect robust differences among treatments consistently. Thus interpretation of the responses observed must be cautious. However, because the serum PRL concentrations of SDPP+PRL cows did not reach that of LDPP cows, it is reasonable to assume that a lower concentration of PRL reached the mammary gland of SDPP+PRL cows compared with LDPP cows, and the previously observed shifts in the PRL signaling cascade would be intermediate as well.

Consistent with the intermediate level of circulating PRL in SDPP+PRL cows, milk yield was intermediate relative to SDPP and LDPP treatment. Greater milk yield in SDPP relative to LDPP cows confirms previous observations that treatment of dry cows with SDPP increases yield in the subsequent lactation [12,17,27]. Further evidence of a relationship between prepartum PRL and subsequent yield comes from the work of Aharoni *et al*. [29], who used mathematical modeling to show that day length prepartum is inversely related to milk production in the next lactation. In general, photoperiodic treatment did not affect milk composition, which is consistent with previous results [12,17]. 

We found a negative correlation between serum PRL concentrations at parturition and milk production through 120 d of lactation. That is, the peak of the periparturient surge PRL concentration was inversely related to subsequent milk yield. During established lactation, PRL is released during milking or suckling, indicating a role for the hormone in the maintenance of milk production [30,31]. There have been several reports describing a positive relationship between PRL concentrations and milk yield during established lactation in various species including rodents [32,33] and rabbits [34]; however, the effects of PRL on milk yield in dairy cows have been variable [35,36,37]. To our knowledge, this is the first report of a direct negative relationship between prepartum serum PRL concentrations and subsequent milk yield, although previous studies of the effect of SDPP during the dry period supported this concept [12,17,27]. This negative relationship is consistent with the concept of an increase in sensitivity of the mammary gland to PRL when serum concentrations are low due to feedback effects on PRL signaling; such inverse relationships between ligand and receptor have been reported for many other hormones [38].

Previously, it was suggested that a portion of the production response of SDPP treated dry cows was due to increased DMI during the dry period, because in earlier studies SDPP cows had greater DMI compared with those on LDPP during the dry period [12,17,27]. Yet in this experiment, SDPP cows did not consume more dry matter during the dry period than LDPP cows. Another study suggests a seasonal effect on the response of DMI to SDPP, as increased DMI during the dry period was not consistently observed in cows subjected to SDPP at different times of the year [39]. In that experiment and the current study, greater milk production was observed in the SDPP cows when compared with LDPP cows but DMI was not different between these groups. Further, there was no difference in DMI observed after calving, although there was limited time for observation of that variable as cows intake was followed for only 42 d. The lack of effect of treatment on indicators of adipose tissue mobilization, NEFA and BHBA, is additional evidence that nutrient intake was not affected by treatment during the dry period. The increase in NEFA after calving in SDPP cows is consistent with that group’s greater milk yield relative to the LDPP and SDPP+PRL cows. Because the effect of SDPP on DMI is not consistent across studies, and NEFA responses were only observed after calving, the evidence suggests that something other than the increased DMI during the dry period is driving the production response in the subsequent lactation. 

We observed an increase in mammary tissue proliferation during the dry period–which agrees with previous reports [2,14], as well as a tendency for an increase in mammary tissue proliferation in SDPP cows relative to LDPP or SDPP+PRL cows on d −20 relative to parturition. This too is consistent with previous observations [14] and supports the concept that there is a window of sensitivity, approximately three to six weeks prior to parturition, wherein changes in photoperiod can influence mammary cell proliferation. The mechanism by which SDPP exposure increases mammary tissue proliferation is not clearly understood; however, as mentioned previously, it may involve treatment differences in PRL sensitivity of the mammary gland since exposure of cows to SDPP increased of expression of PRL-R mRNA compared with LDPP [17]. In addition, a critical threshold in the abundance of PRL-R mRNA must be reached for normal mammary development and lactation to occur in mammary gland of rodents [21] and it is possible that a similar threshold of PRL-R expression in the mammary gland exists in ruminants. Moreover, PRL promotes mammary cell survival by impinging on the insulin-like growth factor (IGF) axis [40], and exposure of cows to SDPP increased expression of IGF-II in the mammary gland [14]. Therefore, it is plausible that the increase in mammary tissue proliferation in cows exposed to SDPP is a result of increased PRL signaling due to greater PRL-R expression, which may influence the IGF axis and increase mitogenic activity in the mammary gland. 

Evidence that PRL signaling responses are associated with photoperiodic manipulation continues to emerge and in general support the contention that decreasing PRL in dry cows is beneficial to future yield. For example, Wall *et al*. [14] found that cows exposed to SDPP during the dry period had decreased epithelial cell apoptosis during the dry period and increased mammary tissue proliferation at d −24 when compared with LDPP treated cows. Further, Wall *et al*. [41] determined that cows exposed to SDPP cows during the dry period have lower expression of suppressors of cytokine signaling (SOCS) relative to LDPP cows. Expression of SOCS are postulated to negatively affect PRL signaling, so a reduction should lead to increased PRL signaling at the mammary gland which may enhance mammary development during the dry period and result in increased milk production in the subsequent lactation. These data support our hypothesis that PRL sensitivity is at least partially driving the response of dry cows to photoperiod treatment and effects on the subsequent lactation. 

Additional evidence that milk yield has an inverse relationship to circulating PRL during the dry period comes from recent work with heat stress manipulation. Relative to cooled cows, those that experience heat stress when dry have increased PRL through parturition, reduced mammary growth, and produce less milk in the subsequent lactation [42,43,44]. Whereas heat stress also impacts other systems that may affect subsequent performance, for example dry matter intake, it is of interest that basal metabolic profiles remain unchanged relative to cooled cows [45]. Thus, similar to photoperiodic manipulation, it appears that metabolic factors do not play a significant role in the subsequent milk yield response of dry cows to temperature variation.

## 5. Conclusions

Cows exposed to SDPP during the dry period produced more milk in the subsequent lactation than those exposed to LDPP, and this response was partially eliminated by supplementation with exogenous PRL. Mammary tissue proliferation during the dry period was increased in SDPP cows relative to LDPP or SDPP+PRL cows. Milk production through 120 d of lactation was negatively correlated with concentrations of PRL in the serum at parturition. The findings of this experiment provide further evidence that SDPP during the dry period elicits an increase in subsequent milk yield, and this may be mediated in part by changes in serum PRL concentrations and consequent mammary tissue proliferation. 
